# Individual computer-assisted 3D planning for placement of auricular prosthesis anchors in combination with an implantable transcutaneous bone conduction hearing device in patients with aural atresia

**DOI:** 10.1007/s00106-022-01190-w

**Published:** 2022-09-09

**Authors:** Ingmar Seiwerth, Sebastian Plößl, Michael Herzog, Sebastian Schilde, Florian Radetzki, Steffen Krämer, Torsten Rahne, Stefan K. Plontke

**Affiliations:** 1 Department of Otorhinolaryngology, Head and Neck Surgery, University Medicine Halle (Saale), Martin Luther University Halle-Wittenberg, Ernst-Grube-Str. 40, 06120 Halle (Saale), Germany; 2 Department of Otorhinolaryngology, Head and Neck Surgery, Martha-Maria Hospital Halle-Dölau, Halle (Saale), Germany; 3 Department of Otorhinolaryngology, Carl Thiem Klinikum, Cottbus, Germany; 4 Department of Orthopedic and Trauma Surgery, University Medicine Halle (Saale), Martin Luther University Halle-Wittenberg, Halle (Saale), Germany; 5 Department of Orthopedic und Trauma Surgery, Brandenburg Medical School Theodor Fontane, Klinikum Dessau, Dessau, Germany; 6 MASK-Anaplastologen GmbH, Leipzig, Germany

**Keywords:** Conductive hearing loss, Hearing aids, Computer simulation, Bone conduction, Auricle

## Abstract

**Background:**

The simultaneous implantation of the Bonebridge (MED-EL, Innsbruck, Austria), a semi-implantable active transcutaneous bone conduction hearing device and anchors for auricular prostheses can be challenging as both implants contain magnets and compete for the narrow space in the designated implantation area.

**Material and methods:**

A preoperative planning tool (virtual surgery) was used with individual 3D computer models of the skull and implants for finding optimal implant positions for both the floating mass transducer (FMT) and the anchors for the auricular prosthesis. The interaction between the magnetic prosthesis anchors and the FMT was measured by means of static magnetic forces. A retrospective data analysis was conducted to evaluate the surgical and audiological outcome.

**Results:**

Between 2014 and 2021, a 3D planning of a simultaneous implantation of the Bonebridge with auricular prosthesis anchors was conducted on 6 ears of 5 patients (3 males, 2 females; age range 17–56 years). The individual preoperative planning was considered very useful for the optimal placement of bone anchors in combination with the Bonebridge. Audiological data showed a clear benefit for hearing 3 months and > 11 months after implantation. No adverse interactions between the magnetic prosthesis anchors and the FMT were observed. In two patients, revision surgery was carried out due to skin inflammation or wound healing problems. No long-term complications were observed 3–5 years after surgery.

**Conclusion:**

Preoperative 3D planning represents a clear benefit for the simultaneous audiological and esthetic rehabilitation using the Bonebridge and anchors for auricular prostheses.

## Background

Since malformations of the outer and middle ear are regularly associated with conductive hearing loss on the affected side [1], cosmetic rehabilitation and audiological rehabilitation are the usual targets for treatment. Depending on the degree of malformation, a cosmetic ear reconstruction with an attempt for audiological rehabilitation by tympanoplasty is generally considered a multistep, complex, and challenging procedure [26, 27]. Implantable electronic hearing devices have become an interesting option for hearing rehabilitation, especially after unsuccessful reconstruction attempts of the external and middle ear.

The simultaneous implantation of percutaneous bone-anchored hearing devices like the Baha system (Cochlear Bone Anchored Solutions, Gothenburg, Sweden) [14] in combination with auricular prosthesis has already been described [10, 11]. Percutaneous implants, however, show a higher complication rate than other bone conduction hearing systems, especially higher risks of wound infections [15, 23].

In 2012, a semi-implantable, active, transcutaneous bone conduction (BC) hearing device, the Bonebridge (BCI 601), was introduced by MED-EL (Innsbruck, Austria; [31]). Since sound information is transferred by induction from the external audio processor to the implantable part, there is no need for permanent skin penetration [18].

Information is transferred by induction from the audio processor to the implant

Due to anatomical varieties, the surgical placement of the active, acoustic energy-transmitting component of the Bonebridge, the floating mass transducer (FMT; 8.7 mm depth, 15.8 mm diameter), can be challenging with respect to bone thickness and position of the dura or sigmoid sinus, especially in small mastoids as seen in children, in cases of malformations, and after previous ear surgery [19, 21]. If necessary, implantation depth can be reduced by the use of BCI Lifts. Since September 2019, a follow-up model (BCI 602) has been available in Europe with a different FMT geometry (4.5 mm depth, 18.2 mm diameter; [16, 34]).

In patients with congenital malformations of the middle and outer ear—often with a history of unsuccessful auricular reconstruction attempts usually during childhood—rehabilitation with implant-retained ear prosthesis remains an option as definitive therapy [9]. These prostheses represent a safe method for long-term fixation, with success rates for implant survival ranging from 95% to 99% [9].

The FMT and the prosthesis anchors compete for a limited surgical field

In cases of a simultaneous audiological rehabilitation with implantation of a transcutaneous, semi-implantable bone-anchored hearing device, the situation can become more complicated, since the FMT and the prosthesis anchors (and their magnets) compete for a limited surgical field. Therefore, optimal planning of placement of the Bonebridge FMT and the prosthesis anchors is important. In this study, a three-dimensional (3D) planning method that was previously developed in our department [17] was extended to the planning of simultaneous placement of auricular prosthesis anchors in combination with the Bonebridge system.

The aim of this study was to evaluate the surgical and functional outcome of the simultaneous placement of anchors for individual ear prosthesis in combination with the Bonebridge system.

## Methods

This retrospective study was conducted with the approval of the responsible ethics committee of the Medical Faculty, Martin Luther University Halle-Wittenberg (No. 2019-123). The retrospective chart review included all patients between January 2014 and November 2021 for whom there was a simultaneous planning of the Bonebridge hearing implant and prosthesis anchors.

### Preoperative planning tool

Based on Digital Imaging and Communications in Medicine (DICOM) data of thin-layer computed tomography (CT) scans, 3D models of the temporal bones were reconstructed by means of the visualization software Amira (versions 5.2–6.3, FEI Visualization Sciences Group, Burlington, MA, USA). In a second step, 3D models of the BC-FMT and the prosthesis anchors were fused with the 3D model of the temporal bone in each patient. Through “virtual surgery” all components could be moved freely and independently until the optimal positions for the FMT and the prosthesis anchors were determined.

**Fig. 1:**
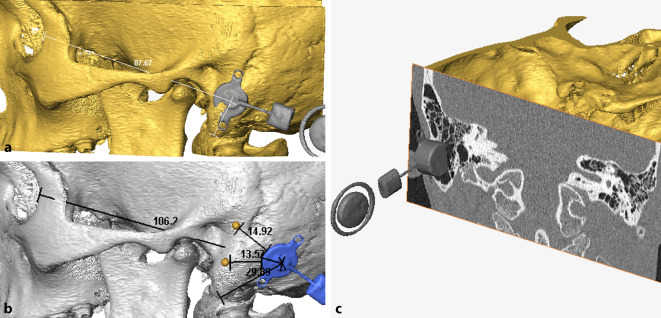
Preoperative planning, demonstrated as an example in patient 1. **a** Optimal placement of the floating mass transducer (FMT) without planning of prosthesis anchors (sinodural angle). **b** 3D planning with prosthesis anchors and retrosigmoidal FMT implant position with marked distances to landmarks and prosthesis anchors for intraoperative transfer. **c** View of the initially planned sinodural angle position with coronal computed tomography scan

**Fig. 2:**
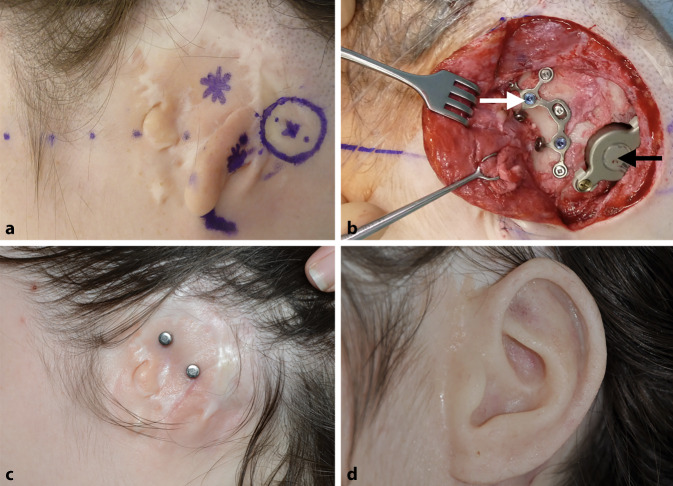
Patient 1. **a** Preoperative marking of the implant positions and the distances to the landmarks. **b** Intraoperative view with the implanted Bonebridge (*black arrow*) and the plate for prosthesis anchors (*white arrow*). **c** Final result after healing period with magnets. **d** Auricular prosthesis in situ

In all 3D image sections, there was the possibility to overlay the model with the respective axial, sagittal, or coronal CT scan layers. Thus, the designated site of the FMT and the prosthesis anchors could be verified from all perspectives with respect to the correct position and possible intracranial impression (dura or sigmoid sinus). For intraoperative identification of the optimal position of the implants in the intraoperative situation, distances from the center of the FMT and the anchors were measured to anatomic landmarks, which in general were the zygomatic root, the mastoid tip, and the lateral orbital margin (Fig. 1b). Intraoperatively, the intersection points of these lines indicated the correct implant and anchor positions (Fig. 2a).

### Surgery

As a first step of the surgical procedure, auricular rudiments or remnants from former surgeries (usually during childhood) were resected if present. The calculated positions of the FMT and the prosthesis anchors were marked according to the preoperative 3D planning tool results (Fig. 1b and 2a). After skin incision, the bed for the FMT was drilled. The dura and the sigmoid sinus might be skeletonized as surgical landmarks. Although a slight impression of these structures is acceptable, we tried to avoid this by means of the preoperative 3D planning. For necessary impressions of more than 1–2 mm, the FMT was elevated by BCI Lifts. After that, prosthesis anchors (Epiplating-system, Medicon, Tuttlingen, Germany) were positioned according to the preoperative 3D planning and fixed. After skin closure, “healing caps” were attached to the anchors and replaced by the magnets after the healing period.

### Anaplastology

Individual ear prostheses were manufactured by an anaplastologist (SK). They consisted of medical grade 2 silicone with hardness degrees of 10–30 shore. The magnets used (steco-system-technik, Hamburg, Germany) were characterized by pull forces between 1.4 and 3 N per magnet.

### Interaction of magnets and audiology

Interaction between the magnets of the prosthesis and the FMT, which also contains a movable magnet for vibration energy transfer to the skull, was measured. Permanent magnets as used with the prostheses were put on the skin at different positions surrounding the FMT within a circle of 4 cm in diameter and at the final positions of the prosthesis anchors. Live-voice stimulation was applied to the audio processor in place. Sound quality was evaluated subjectively by the patient. A reduced stimulation would result in a decrease of maximum output force level and thus in a reduction of the dynamic range or increased distortion.

### Audiological assessment

Pure-tone thresholds for air conduction (AC) and bone conduction (BC) were measured as used in clinical routine practice and reported for the treated side, while warble tones were used for postoperative aided thresholds.

Speech recognition at 65 dB SPL (WRS_65_) in quiet was measured 1–3 months and > 11 months postoperatively using the German Freiburger speech perception test. Speech recognition in noise was evaluated postoperatively with the German Oldenburg matrix test in aided and unaided conditions with presentation of speech and noise from the front (S_0_N_0_).

## Results

### Demographics

Between 2014 and 2021, in six ears and five patients (three males, two females; mean age: 43.6, range: 17–56 years) preoperative 3D planning of ear prosthesis and simultaneous Bonebridge implantation was conducted as “virtual surgery.” The audiological data of some of these patients were reported previously as part of a larger series [24].

All patients had multiple unsuccessful attempts of auricular reconstruction, ear canal reconstructions, or ear surgeries (Table 1). Preoperatively, all patients were discussed in our interprofessional hearing implant board including other options of hearing rehabilitation such as percutaneous bone conduction or active middle ear implants. Due to different individual reasons, for example, anatomical limitations or the patient’s wish, alternative options were deselected in all cases. In one of these patients (patient 3), prosthesis anchors were already in situ and only the FMT of the Bonebridge had to be added at an appropriate position. One patient (patient 5) had simultaneous implantation of a Bonebridge and prosthesis anchors 5 years before at another institution. Due to wound infection of the prosthesis anchors and dislocation of the FMT from the implant bed, preoperative 3D planning was conducted to change the implant to the BCI 602 model.

**Table 1:** Demographics and clinical data

ID	Age at surgery (years)	M/F	Treated side	Diagnosis/indication	Otologic history (treated ear)	FMT-position	Sinus/dura exposure (Bonebridge-implantation)	Complications
1	17	F	L	Malformation (microtia, EAC atresia, incus and malleus dysplasia)	Multiple failed reconstructions of the external ear	RS	Partial exposure of sigmoid sinus and dura, BCI Lifts (2 mm) to avoid impression	None
2	51	M	R	Malformation of external and middle ear, chronic OE, partial tympanic fibrosis, post-inflammatory stapes fixation, chronic mastoiditis	Ear canal surgery; CWD surgery	SDA	Exposure of sinus and minimal impression with 1 mm BCI Lifts	Partial skin necrosis, revision surgery 22 days post-OP
3	45	M	R	Ear malformation; prosthesis anchors already in situ	Ear canal surgery; 2 TPL-revisions; implantation and re-implantation of prosthesis anchors	SDA	Minimal sinus exposure, SDA covering with TachoSil^a^	None
4	49	M	R	Bilateral ear malformation and ear canal atresia, chronic secreting OE	Ear canal surgery and CWD surgery in childhood	SDA	Minimal sinus impression, 2 mm BIC Lifts	Granulation tissue around prosthesis anchor → revision surgery 2 months post-OP
4	49	M	L	Bilateral ear malformation and ear canal atresia, chronic secreting OE	Ear canal surgery and CWD surgery in childhood	SDA	Dura exposed, minimal sinus impression, covering with TachoSil^a^	None
5	56	F	R	FMT-protrusion and dislocation, skin infection around prosthesis anchor, secreting COM, malformation	Ear canal surgery, implantation of Bonebridge and prosthesis anchors 5 years before (alio loco)	Planning only: SDA	N/A	N/A

*BCI* bone conduction implant (BCI 601), *COM* chronic otitis media, *CWD* canal wall down mastoidectomy, *EAC* external auditory canal, *F* female, *FMT* floating mass transducer, *ID* patient identification number, *L* left, *M* male, *N/A* not applicable, *OE* otitis externa, *post-OP* postoperative, *R* right, *RS* retrosigmoidal, *SDA* sinodural angle, *TPL* tympanoplasty

^a^Takeda, Berlin, Germany

### Surgery

**Fig. 3:**
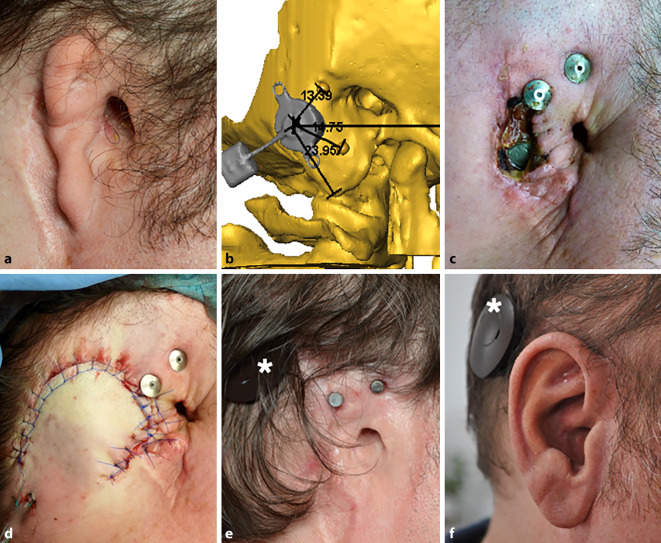
Patient 2. **a** Preoperative situation. **b** Preoperative three-dimensional planning. **c** Wound healing problem with partial skin necrosis in the resection area of auricular rudiments. **d** Revision surgery with rotational flap 22 days postoperatively. **e** Postoperative situation after healing with magnets and sound processor (*asterisk*). **f** Postoperative view with auricular prosthesis and sound processor (*asterisk*)

**Fig. 4:**
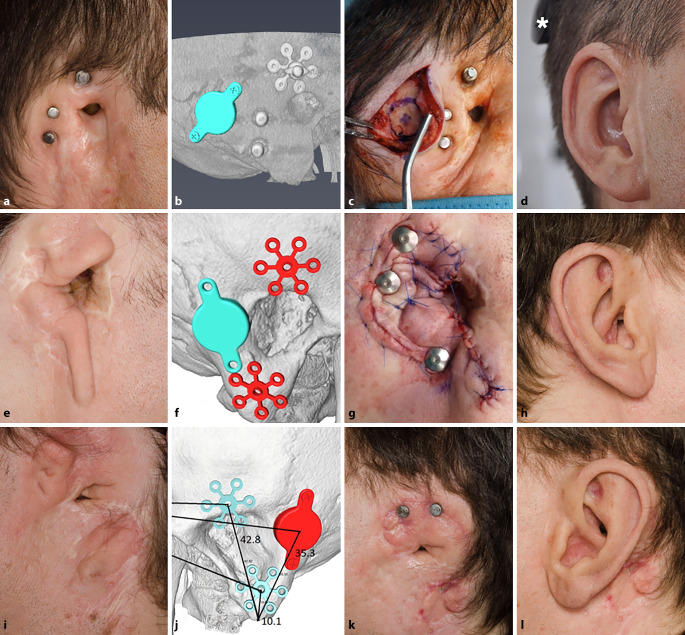
Patient 3 (**a–d**) and patient 4 right (**e–h**) and left (**i–l**). **a,** **e,** **i** Preoperative situation (in the case of patient 3 with already existing prosthesis anchors) **b,** **f,** **j** Preoperative three-dimensional planning. **c,** **g** Intraoperative situation. **k** Postoperative situation. **d,** **h,** **l** Postoperative view with auricular prosthesis and sound processor (*asterisk*)

All Bonebridge implantations were be performed without intraoperative complications (Figs. 2, 3 and 4). In one case, the FMT implantation site needed to be placed in a retrosigmoidal position, as preoperatively planned (patient 1, Fig. 1), while in the other four cases, the FMT was implanted in the sinodural angle (mastoid). In four cases (three patients), prosthesis anchors were simultaneously implanted. The definitive decision on the type of prosthesis anchor (“star” or combined plate) was made intraoperatively, depending on the anatomical situation. Therefore, the final anchor position could deviate from the preoperatively planned position. In one patient (patient 3, Fig. 4), prosthesis anchors were already in situ.

Sinus or dura were exposed in all five Bonebridge implantations. In three of five cases, a minimal impression of sinus or dura was necessary, as expected in the virtual surgery, and in three cases, BCI Lifts (1 or 2 mm) had to be used additionally to avoid a deeper impression of the sigmoid sinus or the dura.

Sinus or dura were exposed in all five Bonebridge implantations

**Fig. 5:**
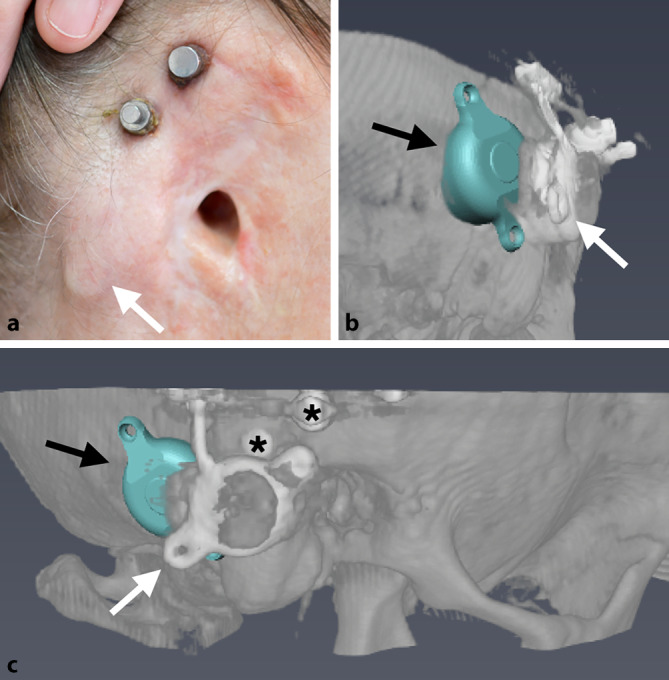
Patient 5. **a** Preoperative view with subcutaneously clearly visible floating mass transducer (FMT) of the BCI 601, dislocated from the bony bed. **b,** **c** Preoperative three-dimensional planning with optimal position of the FMT of the BCI 602 (*black arrows*) dorsal to the dislocated FMT of the BCI 601 (*white arrows*). *Asterisks*: prosthesis anchors

As can be seen in patient 4 on the right side (Fig. 4e–h), the intraoperative situation necessitated implanting two superior anchors by a combined plate instead of one, as initially planned. In the case of patient 5, the optimal implant position (BCI 602) was located dorsal to the current position of the dislocated FMT (BCI 601) considering a safety distance to the prosthesis anchors. The FMT, which was dislocated from the implant bed mainly with its caudal part, was in close proximity to the prosthesis anchors (Fig. 5). Shortly before surgery, the patient declined a new Bonebridge implantation. Therefore, the dislocated Bonebridge BCI 601 was explanted, the dura reinforced, and a wound revision around the prosthesis anchors and an occlusion of the external ear canal with a local flap were performed. Consequently, patient 5 was excluded from audiological analysis. Intraoperatively, the implant was found encapsulated with connective tissue and the FMT was tilted out of the implant bed with the caudal screw outside the bone, as can also be seen in the preoperative planning (Fig. 5). Sinus and dura were partially exposed and overgrown with connective tissue.

Due to a postoperative complication with partial skin necrosis in one patient (patient 2, Fig. 3c), revision surgery with a rotational flap was necessary 22 days after surgery (Fig. 3d). In another patient (patient 4, right side), granulation tissue had to be reduced around the prosthesis anchors 2 months postoperatively. In the same surgery, ear rudiments were removed on the left (contralateral) side, where a simultaneous implantation of the Bonebridge and prosthesis anchors was planned at a later time. In this patient, only the superior anchors were used on both sides for prosthesis attachment due do anaplastological reasons (Fig. 4k). All postoperative complications were successfully resolved, and no long-term-complications (3–5 years after surgery) were observed.

### Interaction of magnets

No adverse interactions between the magnetic prosthesis anchors and the FMT were found. Sound quality did not change with distance to, and thus with the strengths of the potentially disturbing static magnetic field. No magnetic-field-induced changes in hearing were observed whether the prosthesis was in place or not.

### Audiological results

**Fig. 6:**
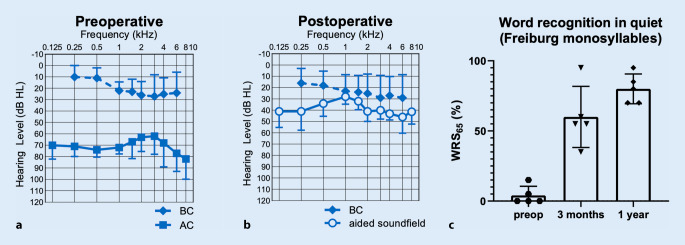
Audiological results (patients 1–4). **a** Mean preoperative pure-tone thresholds for bone conduction and air conduction. **b** Mean postoperative pure-tone thresholds for bone conduction and air conduction (aided sound field) **c** Word recognition score in quiet at 65 dB SPL (*WRS*_*65*_). *AC* air conduction, *BC* bone conduction, *preop* preoperative

Before surgery, the mean pure-tone thresholds (4PTA_0.5–4_ _kHz_) were 70 dB HL (standard deviation [SD] 9) for air conduction and 21 dB (SD 7) for bone conduction. Aided air conduction with the Bonebridge improved to 39 dB HL (SD 5) after 3 months and to 37 dB HL (SD 6) after > 11 months postoperatively (Fig. 6a, b).

Mean word recognition score (WRS_65_) in quite increased from 4% (SD 6) preoperatively unaided or 70% (SD 16) best aided to 60% (SD 20) Bonebridge-aided after 3 months, 80% (SD 10) after > 11 months (Fig. 6c), and 63% (SD 6) after 3–5 years. Speech reception thresholds (SRT) in noise improved from −1.4 dB SNR (SD 3) in the unaided setting to −1.8 dB SNR (SD 3) in the aided situation (Bonebridge).

## Discussion

To the authors’ knowledge, this is the first study reporting a 3D planning method (“virtual surgery”) for simultaneous implantation of a Bonebridge bone conduction hearing system with anchors for auricular prosthesis. One case series presented the surgical treatment with bone-anchored auricular prosthesis and the Bonebridge as a one-step surgery in three patients (four ears; [35]). In that study, the implant position was determined two-dimensionally based on high-resolution CT. Another study described the simultaneous Bonebridge implantation with one-stage auricular reconstruction with a Medpor framework (Medpor, Stryker, Kalamazoo, MI, USA) or as two-stage surgery with autologous material also using 3D preoperative planning [4]. Wang et al. also reported a technique of autologous surgical ear reconstruction and Bonebridge implantation as two-step surgery [32], and Fan et al. described a three-step surgical method [7]. While in the study by Fan et al. 3D preoperative planning was also used [7], Wang et al. performed 2D planning [32].

Preoperative 3D planning can generally increase safety in Bonebridge surgery, and a variety of simpler as well as more complex methods of preoperative computer-assisted planning options have been suggested to find an optimal or at least appropriate position for the FMT of the Bonebridge. A systematic review of preoperative Bonebridge planning methods has recently been presented [25]. Nonetheless, especially in cases of additional bone anchor implantation for auricular prosthesis, an accurate transfer of the designated positions of the anchors and implants should be guaranteed by the selected method. Since the area for implantation must be shared between the anchors and the FMT, the possibilities for safe placement of the FMT are limited. As can be seen in the case of patient 4, the situation was additionally aggravated by the open mastoid cavity after canal wall down surgery in childhood (Fig. 4f). Here, a protrusion of the FMT into the open cavity had to be avoided, as infections could spread from the radical cavity to the implant [3]. Regarding patient 5, it cannot be assessed definitively if there is a relationship between the close anchor–FMT distance, the wound infection around the prosthesis anchors, and the dislocated FMT. In this situation, preoperative 3D planning offered an option for a safe FMT placement, even though, at a later timepoint, this was no longer wished by the patient.

The “competing” situation of prosthesis anchors and the FMT can be attenuated by using smaller bone conduction hearing rehabilitation options such as percutaneous bone-anchored devices or alternatives such as active middle ear implants (aMEI). Percutaneous devices, however, are associated with an additional risk of wound infections [15], and the possibilities for implantation of an aMEI can be limited due to anatomical or clinical reasons. Especially in malformations [12], aMEI can be associated with an increased risk of surgery, for example, a deterioration of the bone conduction threshold [28]. In recent studies, an explantation rate of 10.2% [2] and, depending on the coupling position of the FMT, revision rates between 10.2 and 29% were described [6, 22, 30].

Preoperative 3D planning can increase safety in Bonebridge surgery

From an audiological point of view, the distance of the position of bone stimulation to the cochlea seems to influence hearing thresholds [20]. With regard to prosthesis anchors, Federspil described the ideal position for retention elements at a distance of 2 cm to the center of the outer ear canal [8]. These aspects may improve the functional and surgical outcome and should be considered during 3D planning.

In two patients, revision surgery was indicated due to skin inflammation or wound healing problems, and in one case with partial skin necrosis (Fig. 3c, d). These complications might have been (but were not necessarily) associated with the simultaneous implantation of the bone-anchored hearing system and the prosthesis anchors. Due to the history of—often multiple—previous ear surgeries, and consequently severe scar formation in the surgical field, there was very likely a significantly higher risk of wound-healing problems.

There were no long-term complications in all five ears with Bonebridge and prosthesis anchors

No long-term complications were observed in all five ears with Bonebridge and prosthesis anchors during a follow-up period of 3–5 years after surgery.

No magnetic interaction between the Bonebridge and the prosthesis anchors was observed. All patients showed a relevant functional (audiological) benefit after Bonebridge implantation, which is consistent with previous studies [5, 13, 29, 31, 33].

In September 2019, the follow-up model BCI 602 was introduced in Europe with modified dimensions of the FMT. The implant depth is now reduced to 4.5 mm, and an improvement of bone fit in mastoids of children and young adults was demonstrated [16, 34]. On the other hand, the diameter of the FMT was increased to 18.2 mm, which could probably aggravate the competing situation with the prosthesis anchor positions. Therefore, also with the new BCI 602 model, a careful preoperative 3D planning is recommended in cases of simultaneous implantation with auricular prosthesis anchors.

## Practical conclusion

- The simultaneous implantation of the Bonebridge hearing implant system together with anchors for individual auricular prosthesis is an adequate option for simultaneous cosmetic and audiological rehabilitation.
- Preoperative three-dimensional planning can support the optimal identification of the implantation sites.
- Although local tissue conditions may be challenging due to previous surgeries, the esthetic and audiological results of the combined rehabilitation method can be considered as beneficial.
- No long-term complications were observed in the 3–5-year follow-up period.

## Declarations

All described procedures or retrospective analyses on humans were conducted in accordance with the institutional ethics committee, with national law and with the 1975 Helsinki declaration and its later amendments.

The supplement containing this article is not sponsored by industry.
